# Efficacy of Single-Dose and Triple-Dose Albendazole and Mebendazole against Soil-Transmitted Helminths and *Taenia* spp.: A Randomized Controlled Trial

**DOI:** 10.1371/journal.pone.0025003

**Published:** 2011-09-27

**Authors:** Peter Steinmann, Jürg Utzinger, Zun-Wei Du, Jin-Yong Jiang, Jia-Xu Chen, Jan Hattendorf, Hui Zhou, Xiao-Nong Zhou

**Affiliations:** 1 Department of Epidemiology and Public Health, Swiss Tropical and Public Health Institute, Basel, Switzerland; 2 University of Basel, Basel, Switzerland; 3 National Institute of Parasitic Diseases, Chinese Center for Disease Control and Prevention, Shanghai, People's Republic of China; 4 Helminthiasis Division, Yunnan Institute of Parasitic Diseases, Simao/Pu'er, People's Republic of China; The George Washington University Medical Center, United States of America

## Abstract

**Background:**

The control of soil-transmitted helminth (STH) infections currently relies on the large-scale administration of single-dose oral albendazole or mebendazole. However, these treatment regimens have limited efficacy against hookworm and *Trichuris trichiura* in terms of cure rates (CR), whereas fecal egg reduction rates (ERR) are generally high for all common STH species. We compared the efficacy of single-dose *versus* triple-dose treatment against hookworm and other STHs in a community-based randomized controlled trial in the People's Republic of China.

**Methodology/Principal findings:**

The hookworm CR and fecal ERR were assessed in 314 individuals aged ≥5 years who submitted two stool samples before and 3–4 weeks after administration of single-dose oral albendazole (400 mg) or mebendazole (500 mg) or triple-dose albendazole (3×400 mg over 3 consecutive days) or mebendazole (3×500 mg over 3 consecutive days). Efficacy against *T. trichiura*, *Ascaris lumbricoides*, and *Taenia* spp. was also assessed.

Albendazole cured significantly more hookworm infections than mebendazole in both treatment regimens (single dose: respective CRs 69% (95% confidence interval [CI]: 55–81%) and 29% (95% CI: 20–45%); triple dose: respective CRs 92% (95% CI: 81–98%) and 54% (95% CI: 46–71%)). ERRs followed the same pattern (single dose: 97% *versus* 84%; triple dose: 99.7% *versus* 96%). Triple-dose regimens outperformed single doses against *T. trichiura*; three doses of mebendazole – the most efficacious treatment tested – cured 71% (95% CI: 57–82%). Both single and triple doses of either drug were highly efficacious against *A. lumbricoides* (CR: 93–97%; ERR: all >99.9%). Triple dose regimens cured all *Taenia* spp. infections, whereas single dose applications cured only half of them.

**Conclusions/Significance:**

Single-dose oral albendazole is more efficacious against hookworm than mebendazole. To achieve high CRs against both hookworm and *T. trichiura*, triple-dose regimens are warranted.

**Trial Registration:**

www.controlled-trials.com ISRCTN47375023

## Introduction

Hundreds of millions of people are infected with the common soil-transmitted helminths (STHs), namely hookworms (*Ancylostoma duodenale* and *Necator americanus*), *Ascaris lumbricoides* and *Trichuris trichiura*, many by multiple species concurrently [1]–[5]. *Taenia* spp. infections are also widespread [6], [7]. STHs and taeniasis/cysticercosis belong to the neglected tropical diseases (NTDs) and are responsible for mainly chronic and often inconspicuous morbidity [8], [9]. Iron-deficiency anemia, malnutrition, and impaired physical and cognitive development have all been attributed to STH infections [1], [5], [10]. *Taenia solium* cysticercosis is a major cause of epilepsy and other neurological disorders in developing countries [11], [12].

The current strategy for STH control in highly endemic areas focuses on morbidity control through large-scale administration of single-dose anthelminthics to at-risk populations, particularly school-aged children [9], [13], [14]. Due to the zoonotic nature of taeniasis/cysticercosis, its control must also include the veterinary sector [6], [15]–[17]. At present, only four drugs are recommended by the World Health Organization (WHO) for treating STH infections [13], [18]. The global STH control relies on two of them – albendazole and mebendazole – both benzimidazole carbamates. Albendazole [19] and mebendazole [20] display a broad spectrum of activity and are administered orally, usually at a single dose of 400 mg and 500 mg, respectively [13], [18], [21]. Children below the age of 1 year and pregnant women in the first trimester of pregnancy are not eligible for treatment [13].

Albendazole and mebendazole have been extensively used worldwide for more than 30 years, both as stand-alone treatments and, more recently, in combination with other drugs, e.g., praziquantel (against schistosomiasis and food-borne trematodiasis) or ivermectin (against lymphatic filariasis) [9], [22]–[24]. Surprisingly though, only few clinical trials compared the efficacy of albendazole and mebendazole against STHs. Rather, availability, cost, drug donation programs, and policy instead of the local parasite spectra and evidence determine the choice of which anthelminthic drug is deployed. Justification for the indiscriminate use of either drug is derived from high egg reduction rates (ERRs) achieved with both albendazole and mebendazole, and the assumption that morbidity is a function of infection intensity [25], [26]. However, a recent meta-analysis of randomized placebo-controlled single-dose drug efficacy trials pointed to a marked superiority of albendazole over mebendazole against hookworm, high efficacy (in terms of cure rate [CR]) of both drugs against *A. lumbricoides*, and disappointing efficacy of either drug against *T. trichiura* [18]. Few data are available regarding ERRs.

The aim of this randomized controlled trial was to assess the efficacy of standard single-dose *versus* triple-dose oral albendazole and mebendazole against hookworm and other STH infections in a highly endemic but virtually benzimidazole-naïve population in the People's Republic of China (P.R. China).

## Methods

The protocol for this trial and the supporting CONSORT checklist are available as supporting information; see Protocol S1 and Checklist S1.

### Study Area, Study Period, and Participants

The study was conducted between October and December 2008 in Nongyang, a village located in Menghai county, Yunnan province, P.R. China. Details of the study area, population and epidemiological characteristics, including the prevalence of STHs, *Taenia* spp., and intestinal protozoa, have been described before [3], [27], [28]. The local prevalence of each *A. lumbricoides*, hookworm, and *T. trichiura* exceeded 85% in a survey conducted in 2006 [3]. Upon completion of the 2006 survey, compound mebendazole (mebendazole 100 mg/tablet+levamisole hydrochloride 25 mg/tablet, 2 tablets per day for 3 consecutive days) was distributed to the village population. No further interventions took place until the present study.

### Ethics

The study was approved by the Ethics Committee of Basel (no. 294/08) and the Academic Board of the National Institute of Parasitic Diseases, Chinese Center for Disease Control and Prevention in Shanghai (no. 2008091701). The trial was registered with Current Controlled Trials (identifier: ISRCTN47375023). The study objectives and procedures were discussed with the village head, village committee, and local health care officials who informed the residents. Individuals who were interested to participate signed an informed consent form in Chinese (parents or legal guardians in case of minors aged 5–17 years). Upon study completion, albendazole was provided for treatment of study participants found to be infected at evaluation, drop-outs, sick individuals upon recovery, and pregnant women once beyond the first trimester.

### Interventions, Trial Medication, and Outcome Measures

The trial was designed as a community-based open-label, outcome assessors-blinded randomized controlled trial with four arms: (i) single-dose albendazole (400 mg), (ii) single-dose mebendazole (500 mg), (iii) triple-dose albendazole (3×400 mg, given over 3 consecutive days), and (iv) triple-dose mebendazole (3×500 mg, given over 3 consecutive days). No placebo drugs were given to individuals assigned to single dose treatment (open label).

Albendazole (Zentel®; lot no 08060407) was commercially obtained from Sino-American Tianjin SmithKline and French Laboratories Ltd., a Chinese joint venture of GlaxoSmithKline Plc. Mebendazole (Vermox®; lot nos. 8CL4F00 and 7CL8900), produced by Johnson & Johnson/Janssen-Cilag S.p.A., was provided by the WHO regional office in Hanoi, Vietnam.

The primary outcome considered was CR against hookworm 3–4 weeks following dosing. Changes in hookworm infection intensity, as determined by ERR, and efficacy against *A. lumbricoides* and *T. trichiura* served as secondary outcomes. Additionally, the effects of all four treatment regimens on *Taenia* spp. were assessed.

### Eligibility Criteria and Sample Size

Eligible for inclusion were all residents of Nongyang aged 5 years and above. The following exclusion criteria were applied: presence of diagnosed or perceived chronic disease or other conditions likely to interfere with anthelminthic treatment (e.g., hypersensitivity to anthelminthics), pregnancy (verbally assessed at enrolment and again before treatment), recent history of anthelminthic treatment, and participation in other trials (within 1 month).

The intended sample size at enrolment was 370 individuals, based on the following assumptions: a total of 176 individuals (44 in each of the four treatment arms) would be needed to detect differences in the CR following different treatments for the cure of hookworm infections with 80% power using a 2-sided statistical test with an α-level of 0.05 and CRs of albendazole and mebendazole against hookworm infections of 75% and 45%. According to Keiser and Utzinger [18], the respective CRs are 78% and 23%; the higher estimate for the CR of mebendazole was employed in order to include a safety margin. The local prevalence of hookworm infections was assumed to be 60% and compliance was estimated to be 80%. Recruitment was to be stopped once 400 individuals had been enrolled.

### Field and Laboratory Procedures

Families were contacted in batches of 20–30 (∼80–120 potential participants) based on family registry numbers. Interested family members were invited to the local primary school for further information and enrolment. No monetary compensation was offered for participation. Participants answered a short questionnaire investigating demographic and health-related issues, and were given a stool collection container labeled with a unique identifier and their full name. The ability of all study participants to recognize their collection container was determined, and the importance of using the own receptacle emphasized. Each morning, filled containers were collected, and a new container handed out with the aim to obtain two stool samples from each participant.

Stool samples were forwarded to a nearby laboratory and processed on the collection day. First, samples were visually inspected for adult *A. lumbricoides* and *Taenia* spp. proglottids. Second, two 41.7 mg Kato-Katz thick smears [29] were prepared from each sample. Depending on the ambient temperature and considering over-clearance of hookworm eggs, slides were read within 30–90 min of preparation [30]. At least 5% of the daily diagnoses were cross-checked by the principal investigator. Procedures for the evaluation of the treatment efficacy commenced 3 weeks post-treatment, lasted 2 weeks, and involved all participants given at least one drug dose. The same approach was adhered to as during the baseline survey.

### Randomization

All participants who had submitted at least one stool sample during the baseline survey were randomly assigned either to the albendazole or the mebendazole arm of the study. In an independent randomization step, single or triple dose treatment using two computer-generated random sequences of 0 and 1 which were aligned with the list of participants in ascending order of their identification numbers. The eligible individuals were neither stratified by age nor sex before randomization.

### Drug Administration

For each day of treatment, an envelope of the type locally used to hand out drugs was labeled with the name, identification number, and number of treatment, loaded with the appropriate drugs, and sealed. The distribution teams directly observed drug intake after asking about acute health problems and pregnancy status. Study participants had been reminded not to drink alcohol on treatment days and to report emerging health problems to the study physician (a medical doctor from a nearby hospital who visited the village each morning after drug distribution), any member of the research team, or the head of the village. On the second morning – 36 hours after the first dosing – all participating households were visited and participants actively solicited to report any potential adverse events. Reported health problems were classified by the study physician and graded by severity according to a pre-defined scale.

### Statistical Analysis

Data were double-entered in EpiData version 3.1 (EpiData Association; Odense, Denmark) or Microsoft® Excel 2002 (Microsoft; Redmond, USA). After removing discrepancies, the datasets were aligned, and the accuracy of the merged database verified against the original data through random cross-checking. All analyses were performed on a per-protocol basis. Only participants with complete datasets were included.

Baseline and post-treatment prevalences were estimated, and CRs determined for each study arm. The extent of prevalence reductions and differences in CRs between groups were explored, using a 2-sided 2-sample test of proportions, which tests the equality of proportions using large-sample statistics. For each participant, the species-specific helminth infection intensity at baseline and at treatment evaluation was calculated and expressed as eggs per gram of stool (EPG), based on the arithmetic mean of the quadruplicate Kato-Katz thick smear readings, multiplied by a factor 24. Arithmetic and geometric means and ERRs were calculated according to Montresor et al. [31]. Confidence limits for the ERR were calculated using a bootstrap re-sampling method with 2000 iterations. Significant treatment group differences were defined by non-overlapping 95% confidence limits. For all tests, a *p*-value of 0.05 was considered the limit of statistical significance, and 95% confidence intervals (CIs) were calculated as appropriate. Statistical analyses were done in STATA version 10.1 (StataCorp LP; College Station, USA), bootstrap confidence intervals were calculated using R 2.9.1.

## Results

### Participant Flow and Baseline Characteristics

**Figure 1:**
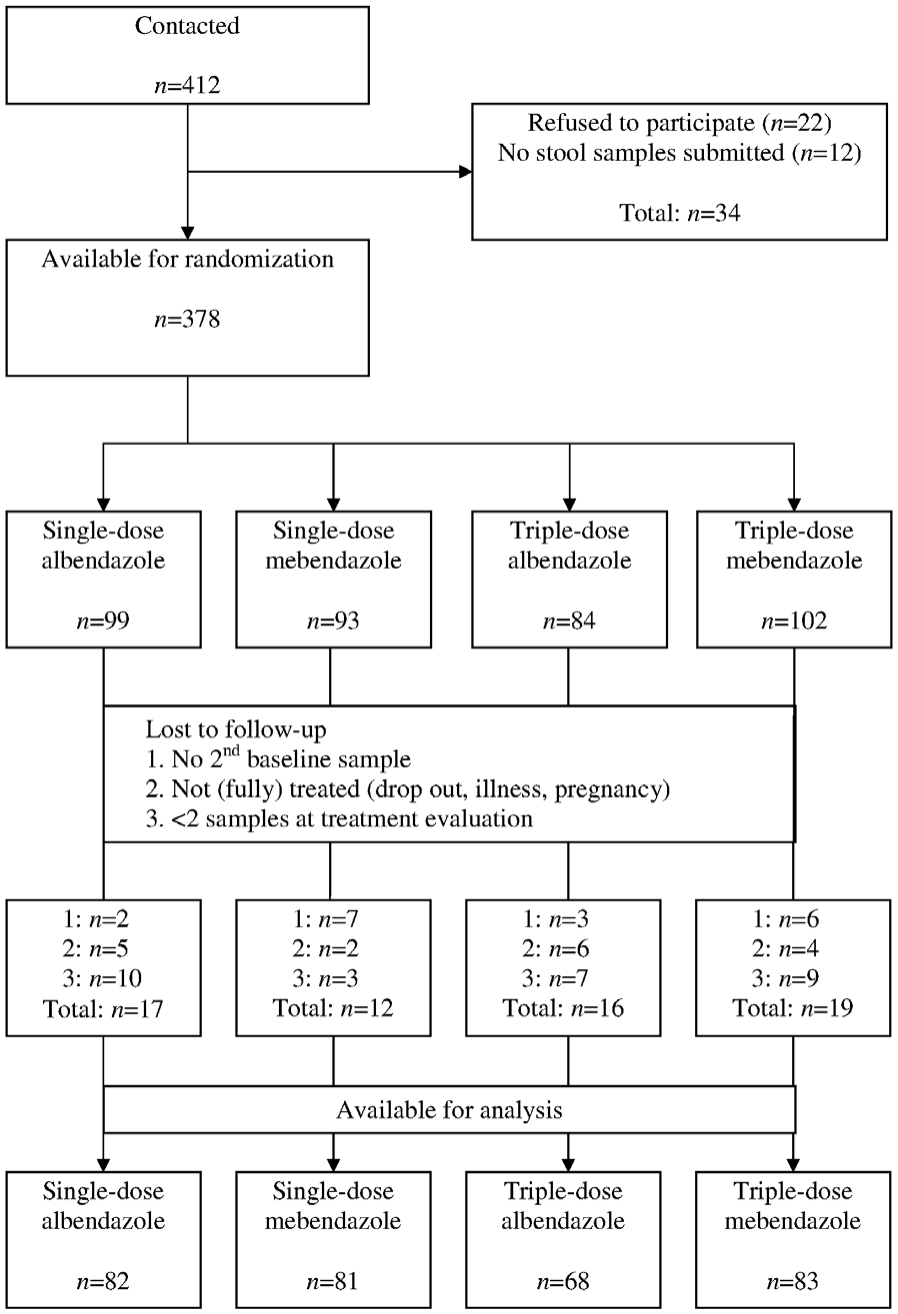
Participation and drop-out at various stages in a trial assessing the efficacy of anthelminthic drugs. Participation and causes for drop-out at various stages in a randomized controlled trial assessing the efficacy of single-dose *versus* triple-dose albendazole and mebendazole against STH infections and *Taenia* spp. in a Bulang ethnic minority community in Yunnan province, P.R. China in late 2008.

As detailed in Figure 1, at least one stool sample was available from 378 people who were randomly assigned to one of the four treatment arms. Among them, 314 (83%) could be included in the final analysis. The composition of all four groups with regard to sex and age was comparable and baseline prevalences of *A. lumbricoides*, *T. trichiura*, hookworm and *Taenia* spp. were 90%, 75%, 73% and 11%, respectively, with no differences among the four treatment arms (Table 1).

**Table 1:** Demographic characteristics of the participants in a trial assessing the efficacy of anthelminthic drugs.

	Total	Single-dose albendazole	Single-dose mebendazole	Triple-dose albendazole	Triple-dose mebendazole
Total n (%)	314 (100)	82 (100)	81 (100)	68 (100)	83 (100)
Sex: Female n (%)	151 (48.1)	35 (42.7)	39 (48.2)	36 (52.9)	41 (49.4)
Age n (%)					
- 5–14 years	42 (13.4)	9 (11.0)	8 (9.9)	14 (20.6)	11 (13.3)
- 15–24 years	88 (28.0)	26 (31.7)	20 (24.7)	19 (27.9)	23 (27.7)
- 25+ years	184 (58.6)	47 (57.3)	53 (65.4)	35 (51.5)	49 (59.0)
Parasite n (%)					
- Hookworm (95% CI)	228 (72.6; 67.7–77.5)	55 (67.1)	58 (71.6)	50 (73.5)	65 (78.3)
*- Ascaris lumbricoides* (95% CI)	284 (90.4; 87.2–93.7)	78 (95.1)	71 (87.7)	63 (92.6)	72 (86.7)
*- Trichuris trichiura* (95% CI)	234 (74.5; 69.7–79.3)	65 (79.3)	63 (77.8)	48 (70.6)	58 (69.9)
*- Taenia* spp. (95% CI)	33 (10.5; 7.1–13.9)	10 (12.2)	6 (7.4)	7 (10.3)	10 (12.0)

Demographic characteristics and baseline helminth prevalence of the study participants in a randomized controlled trial assessing the efficacy of single-dose and triple-dose albendazole *versus* mebendazole against STH infections and *Taenia* spp. in a Bulang ethnic minority community in Yunnan province, P.R. China, stratified by treatment arm.

### Efficacy Against Hookworm and Other STHs

A single dose of albendazole cured 69% (95% CI: 55–81%) of the hookworm infections, while single-dose mebendazole only cured 31% (95% CI: 20–45%), significantly less (Table 2 and table 3). Triple doses of either drug were significantly more efficacious than single-dose regimens, but the difference between the two drugs persisted: triple-dose albendazole cured significantly more hookworm infections (92%, 95% CI: 81–98%) than triple-dose mebendazole (58%, 95% CI: 46–71%).

**Table 2:** Prevalences and cure rates in a trial assessing the efficacy of anthelminthic drugs (hookworm and *Ascaris lumbricoides*).

	Single-dose albendazole (*n* = 82)	Single-dose mebendazole (*n* = 81)	Triple-dose albendazole (*n* = 68)	Triple-dose mebendazole (*n* = 83)
**Hookworm**				
Prevalence at baseline [% (n)]	67.1 (55)	71.6 (58)	73.5 (50)	78.3 (65)
Prevalence after treatment [% (n)]	20.7 (17)	50.6 (41)	5.9 (4)	36.1 (30)
New positives at evaluation	0	1	0	3
Cure rate [% (95% CI)]^excluding new positives at evaluation^	69.1 (55.2–80.9)	31.0 (19.5–44.5)	92.0 (80.8–97.8)	58.5 (45.6–70.6)
Difference between drug-specific cure rates [% (95% CI)]	38.1 (21.0–55.1)***	Reference	33.5 (19.4–47.7)***	Reference
Difference single- vs. triple-dose cure rates [% (95% CI)]	Reference	Reference	22.9 (8.6–37.2)**	27.4 (10.5–44.3)**
***Ascaris lumbricoides***				
Prevalence at baseline [% (n)]	95.1 (78)	87.7 (71)	92.6 (63)	86.7 (72)
Prevalence after treatment [% (n)]	3.7 (3)	6.2 (5)	2.9 (2)	6.0 (5)
New positives at evaluation	0	0	0	0
Cure rate [% (95% CI)]^excluding new positives at evaluation^	96.1 (89.1–99.2)	93.0 (84.3–97.7)	96.8 (89.0–99.6)	93.1 (84.5–97.7)
Difference between drug-specific cure rates [% (95% CI)]	3.2(−4.1–10.5)	Reference	3.8 (−3.5–11.1)	Reference
Difference single- vs. triple-dose cure rates [% (95% CI)]	Reference	Reference	0.7 (−5.4–6.8)	0.1 (−8.2–8.4)

Cure rates following single-dose and triple-dose albendazole *versus* mebendazole against STH infections and *Taenia* spp., and comparisons between treatment arms. * P value<0.05, ** P value<0.01, *** P value<0.001.

**Table 3:** Prevalences and cure rates in a trial assessing the efficacy of anthelminthic drugs (*Trichuris trichiura* and *Taenia* spp.).

	Single-dose albendazole (*n* = 82)	Single-dose mebendazole (*n* = 81)	Triple-dose albendazole (*n* = 68)	Triple-dose mebendazole (*n* = 83)
***Trichuris trichiura***				
Prevalence at baseline [% (n)]	79.3 (65)	77.8 (63)	70.6 (48)	69.9 (58)
Prevalence after treatment [% (n)]	53.7 (44)	49.4 (40)	32.4 (22)	25.3 (21)
New positives at evaluation	1	2	1	4
Cure rate [% (95% CI)]^excluding new positives at evaluation^	33.8 (22.6–46.6)	39.7 (27.6–52.8)	56.2 (41.2–70.5)	70.7 (57.3–81.9)
Difference between drug-specific cure rates [% (95% CI)]	−5.8 (−22.5–10.8)	Reference	−14.4 (−32.7–3.8)	Reference
Difference single- vs. triple-dose cure rates [% (95% CI)]	Reference	Reference	22.4 (4.3–40.5)*	31.0 (14.2–47.8)***
***Taenia*** ** spp.**				
Prevalence at baseline [% (n)]	12.2 (10)	7.4 (6)	10.3 (7)	12.0 (10)
Prevalence after treatment [% (n)]	7.3 (6)	4.9 (4)	0 (0)	1.2 (1)
New positives at evaluation	1	1	0	1
Cure rate [% (95% CI)]^excluding new positives at evaluation^	50.0 (18.7–81.2)	50.0 (11.8–88.2)	100 (59.0–100)	100 (69.2–100)
Difference between drug-specific cure rates [% (95% CI)]	0 (NA)	Reference	0 (NA)	Reference
Difference single- vs. triple-dose cure rates [% (95% CI)]	Reference	Reference	50.0 (19.0–80.1)*	50.0 (10.0–90.0)*

Cure rates following single-dose and triple-dose albendazole *versus* mebendazole against STH infections and *Taenia* spp., and comparisons between treatment arms. * P value<0.05, ** P value<0.01, *** P value<0.001.

Triple-dose mebendazole exhibited the highest reduction in *T. trichiura* prevalence (CR: 71%), followed by triple-dose albendazole (56%). Single dose applications were found to be significantly less efficacious (mebendazole: 40%, albendazole: 34%). In both cases, the differences between drug-specific CRs were not statistically significant. As expected, both albendazole and mebendazole cleared most of the *A. lumbricoides* infections with observed CRs ranging between 93% and 97%. The efficacies of albendazole and mebendazole were comparable. Triple-dose treatment tended to be slightly more efficacious than single-dose treatment, but the difference was not statistically significant. For *Taenia* spp., a single dose of either drug cured about one half of the infections; triple-dose administration cured all infections.

Table 4 and 5 (and in greater detail the Figure S1) show the baseline EPGs and changes following treatment. In general, the efficacy regarding ERRs followed a similar pattern as that of CRs. Albendazole outperformed mebendazole in terms of hookworm ERR, whereas mebendazole tended to be more efficacious against *T. trichiura*. Triple-dose regimens exhibited significantly higher ERRs against both parasites. All treatments resulted in ERRs>99.9% against *A. lumbricoides*. The median hookworm egg count in the 228 infected participants was 84 EPG at baseline and 30 EPG in those 92 still infected after treatment. The administration of three doses of albendazole resulted in the highest ERR against hookworm (99.7%; 95% CI: 99–99.9%). Single-dose albendazole with an ERR of 97% (95% CI: 95–99%) performed as well as triple-dose mebendazole (96%, 95% CI: 93–98%). A single dose of mebendazole resulted in an ERR of only 84% (95% CI: 73–90%). For *T. trichiura*, the administration of triple doses resulted in an ERR of 97% for mebendazole, and 94% for albendazole. With ERRs of 83% and 77%, respectively, single doses performed significantly worse.

**Table 4:** Infection intensity and egg reduction rates in a trial assessing the efficacy of anthelminthic drugs (geometric mean).

	Single-dose albendazole	Single-dose mebendazole	Triple-dose albendazole	Triple-dose mebendazole
**Hookworm [n]**	55	58	50	65
EPG at baseline (geometric mean)	69	73	90	86
EPG after treatment (geometric mean)	2	12	0.3	3
ERR; difference in geometric mean [%; (95% CI)]	97.3 (95.2–98.7)^b^	83.6 (72.9–90.3)^a^	99.7 (99.1–99.9)^c^	96.4 (93.3–98.2)^b^
***Ascaris lumbricoides*** ** [n]**	78	71	63	72
EPG at baseline (geometric mean)	8,442	7,855	6,485	8,435
EPG after treatment (geometric mean)	0.1	0.5	0.2	0.2
ERR; difference in geometric mean [%; (95% CI)]	>99.9 (>99.9–100)^a^	>99.9 (>99.9–>99.9)^a^	>99.9 (>99.9–100)^a^	>99.9 (>99.9–>99.9)^a^
***Trichuris trichiura*** ** [n]**	65	63	48	58
EPG at baseline (geometric mean)	58	47	68	55
EPG after treatment (geometric mean)	14	8	4	1
ERR; difference in geometric mean [%; (95% CI)]	76.7 (62.6–86.1)^a^	82.5 (71.0–89.6)^a,b^	94.0 (89.4–96.8)^b,c^	97.3 (94.9–98.8)^c^

Infection intensities among those infected at baseline expressed as EPG and ERR following single-dose and triple-dose albendazole *versus* mebendazole against STH infections, and comparisons between treatment arms. Different letters (a, b, c) designate significant differences of ERR between treatment arms, defined by non-overlapping 95% confidence limits (calculated by bootstrap resampling).

**Table 5:** Infection intensity and egg reduction rates in a trial assessing the efficacy of anthelminthic drugs (arithmetic mean).

	Single-dose albendazole	Single-dose mebendazole	Triple-dose albendazole	Triple-dose mebendazole
**Hookworm [n]**	55	58	50	65
EPG at baseline [median (25%–75%)]	78 (30–180)	84 (36–180)	84 (30–210)	102 (30–216)
EPG after treatment [median (25%–75%) [n]]	30 (12–43) [17]	30 (18–126) [41]	36 (18–56) [4]	18 (12–72) [30]
***Ascaris lumbricoides*** ** [n]**	78	71	63	72
EPG at baseline [median (25%–75%)]	9600 (3,576–24,504)	10,260 (4,476–18,744)	8736 (2,382–22,056)	7956 (4,608–19,050)
EPG after treatment [median (25%–75%) [n]]	18 (6–396) [3]	1488 (24–2,904) [5]	384 (18–750) [2]	6 (6–6) [5]
***Trichuris trichiura*** ** [n]**	65	63	48	58
EPG at baseline [median (25%–75%)]	66 (24–138)	48 (18–144)	78 (36–132)	51 (18–138)
EPG after treatment [median (25%–75%) [n]]	48 (18–144) [44]	39 (18–57) [40]	30 (18–78) [22]	18 (6–30) [21]

Infection intensities among those infected at time of observation expressed as EPG and ERR following single-dose and triple-dose albendazole *versus* mebendazole against STH infections, and comparisons between treatment arms.

### Adverse Events

Thirteen study participants (4.1%) reported between one and five adverse events following drug administration, mostly in the morning of the third drug distribution day (about 12 hours after the administration of the second dose, if given) and upon active questioning. Four of these individuals were treated with a single dose (3 with mebendazole, 1 with albendazole) while the remaining nine were treated with triple mebendazole (*n* = 5) or triple albendazole (*n* = 4). One symptom was reported by nine individuals, two symptoms by two individuals (1 treated with triple albendazole, 1 with triple mebendazole), three symptoms by one individual (triple mebendazole) and five symptoms by one individual (triple mebendazole). Adverse events included headache (*n* = 3; all mebendazole), abdominal cramps (*n* = 3; 2 mebendazole, 1 albendazole) and the closely related “full stomach” (*n* = 2; mebendazole), and waist pain (*n* = 1; albendazole). Two individuals each reported vomiting, including production of *A. lumbricoides* worms (1 albendazole, 1 mebendazole), diarrhea (2 mebendazole), fatigue (1 albendazole, 1 mebendazole), and chills (2 mebendazole). Vertigo (albendazole), throat pain (albendazole), fever (mebendazole), and a swollen face (mebendazole) were each reported once. None of the study participants requested medical interventions as adverse events were mild and self-limiting. More women than men reported adverse events (ten women among whom four treated with albendazole and six treated with mebendazole *versus* three men; *P* = 0.046) but there was no significant association between the report of adverse events and age, drug, or number of treatments according to the Fisher's exact test.

## Discussion

This randomized controlled trial comparing the efficacy of single and triple dose albendazole and mebendazole confirmed that single oral albendazole is more efficacious than mebendazole against hookworm infections [18], [32]. It also corroborated that triple-dose regimens result in significantly higher CRs than recommended and widely used single-dose regimens [13], [33]. A single dose of mebendazole only cured 31% of the hookworm infections, while the highest CR, after triple albendazole, was 92%. Even triple administration of mebendazole was less efficacious than a single dose of albendazole. Keiser and Utzinger's meta-analysis [18] estimated a CR of only 15% after single-dose mebendazole, and a value comparable to that found in the present study after single-dose albendazole (present study: 69%, meta-analysis: 72%). With regard to ERRs, all four drug regimens resulted in significant reductions among those infected at baseline. A triple dose of mebendazole was significantly more efficacious than a single dose.

The number of *T. trichiura* infections in each treatment arm was significantly, though only moderately reduced, in line with previous findings [18], [33], [34]. As expected, triple doses resulted in higher CRs than a single dose regardless of the drug. Worryingly, the highest CR observed was only 71% following triple-dose mebendazole. Single and triple doses of mebendazole resulted in higher ERRs than the respective number of albendazole administrations. With regard to *A. lumbricoides* infections, high CRs were observed for both drugs even at a single dose; observations that are in line with systematic reviews and meta-analysis [18], [33].

Attention was paid to enhance the sensitivity of STH diagnosis by examining multiple Kato-Katz thick smears before and after drug administration [3], [35], [36]. The low number of “new” infections found at treatment evaluation (Table 2 and table 3) indicates that a high sensitivity had been achieved despite the rather low density of hookworm and *T. trichiura* eggs. Because of the low *Taenia* spp. prevalence and since the study was not designed to evaluate treatment efficacy against this parasite, the respective results should be interpreted with caution. The conventional indicator for the successful cure of *Taenia* spp. infections – i.e., recovery of the scolex – is no definitive proof whenever individuals harbor several worms, and is difficult to perform outside an institutional setting. We focused on the presence of proglottids and eggs.

An open-label trial design was adhered to due to the complexities and high cost for implementing a double-blind trial in a field setting. We are confident that this did not negatively impact on the validity of the results since outcome assessors were blinded. One individual assigned to the triple albendazole group switched to the single-dose group, and in two instances the drug assignment was changed between members of the same family due to an initial mix-up. We used logistic regression to assess if our results were sensitive to the potential effect modifiers age and sex. Age was treated as a categorical variable (categories as in Table 1) and also as a continuous variable (in years). None of the analyses showed noteworthy differences between the crude and adjusted models with respect to the point estimates or CIs of the odds ratios. The sole exception was the treatment regimen (single dose *versus* triple dose) for which adjustment for sex and age showed stronger effects for both drugs in the case of *T. trichiura*.

The susceptibility of the two human hookworm species to albendazole is known to be unequal, with CRs for the more pathogenic *A. duodenale* higher than that for *N. americanus* [37]. Both hookworm species are endemic in P.R. China but the locally predominant species probably is *N. americanus* according to a polymerase chain reaction (PCR)-based [38] species identification performed in a neighboring area [39]. Multiple-species intestinal helminth infections are common [4] but no associations between species have been found and the high prevalence of multiparasitism in the study population is unlikely to diminish the validity of the findings for other settings.

Two additional observations are worth discussing. First, the *A. lumbricoides* CR did not differ significantly (p>0.05) between infection-intensity classes as defined by WHO [40]. Second, the baseline prevalence of *A. lumbricoides* and hookworm was higher among females than males. At evaluation, the difference persisted for hookworm, but had disappeared for *A. lumbricoides*, probably owing to the high CR against the latter parasite. In the case of *T. trichiura*, comparable prevalences were found for males and females at baseline, but treatment with either drug reduced the prevalence in males more markedly than in females.

The raw data of our randomized controlled trial is provided as supplementary files (Data S1 and Codes S1). In the spirit of trial registration prior to conducting clinical research, of open-access publishing, and of evidence-based medicine, we believe that others might find our data useful (e.g. for subsequent meta-analysis of drugs used against STHs). We hope that other clinical investigators and research groups will follow our example.

In conclusion, single-dose albendazole and mebendazole are highly efficacious against *A. lumbricoides*, albendazole is superior to mebendazole for treating hookworm, and mebendazole slightly outperforms albendazole with regard to treating *T. trichiura*. To achieve high CRs against hookworm and *T. trichiura* infections, triple dose regimens should be considered. Yet, for *T. trichiura*, even triple doses only resulted in the cure of a bit more than half of the infections, a result corroborating previous reports [33], [41]. Triple-dose treatment is commonly deemed unfeasible in the context of large-scale drug administration programs based on logistical and organizational considerations [42], an issue which needs careful attention. To justify rolling out triple dose treatment, the additional efforts and costs required to do so must be weighed against the benefit, i.e., the higher treatment efficacy, and hence the prevention of harm. From a patient perspective, triple dose treatment appeared acceptable in the present study. Our findings therefore underscore the need for discovery and development of novel drugs for the management of trichuriasis [21], [33]. Until new drugs become available, it is recommended to investigate ways to boost the efficacy of existing anthelminthics, including combination therapy (e.g., albendazole or mebendazole plus ivermectin) [21], [33], [34] and multiple dosing [21], [33]. The higher efficacy of triple doses for treating *Taenia* spp. infections further tips the balance in favor of triple dose schedules in certain areas. With regard to large-scale interventions, the present results call for a more nuanced approach than the standard single-dose mono-drug distribution. Indeed, our findings emphasize the need for careful assessment of the locally endemic STHs, and the adaptation of the employed anthelminthic drug regimens to the prevailing situation. In populations primarily parasitized by *A. lumbricoides* and/or hookworm infections, single or – in case of a high prevalence or high-intensity hookworm infections – triple-dose albendazole might suffice. Mebendazole treatment with one or better three doses should be adopted in areas with a high prevalence of *T. trichiura* (and possibly *A. lumbricoides*), but a lower number of hookworm infections. In areas where all three species are co-endemic, alternation between albendazole and mebendazole as well as co-administration of different anthelminthic drugs should be considered.

## Supporting Information

Protocol S1 **Trial protocol.**
(DOC)

Checklist S1 **CONSORT checklist.**
(DOC)

Figure S1 **Frequency distribution of baseline EPGs and changes following treatment.**
(PNG)

Data S1 **Raw data of the trial.**
(XLS)

Codes S1 **Codes to raw data of the trial.**
(DOC)
