# Supplementary material for: Efficacy of Single-Dose and Triple-Dose Albendazole and Mebendazole against Soil-Transmitted Helminths and Taenia spp.: A Randomized Controlled Trial
Source: PLoS One. 2011 Sep 27;6(9):e25003. doi: 10.1371/journal.pone.0025003 (PMC3181256; doi:10.1371/journal.pone.0025003)
Supplement: Protocol S1 — Trial protocol. (DOC) [file pone.0025003.s001.doc]

**Study protocol** (Version 1)

**Title**

Safety and efficacy of single-dose and triple-dose albendazole and mebendazole against soil-transmitted helminth infections in humans: open-label randomized-controlled trial in China

**Institutions**

1. Swiss Tropical Institute (STI), Department of Public Health and Epidemiology,
   CH-4051 Basel, Switzerland
2. National Institute of Parasitic Diseases (IPD), Chinese Center for Disease Control and Prevention (China CDC), Shanghai 200025, People’s Republic of China

**Appendices**

1. Study information and informed consent sheet
2. Participation monitoring sheet
3. Adverse event grading and registration sheet

**Basel, 09.09.2008 _________________________ __________________________**

**Prof. Dr. med. Christoph Hatz Prof. Dr. Jürg Utzinger**

**Investigators (CVs enclosed)**

**Dr. Peter Steinmann (Epidemiologist; Principal Investigator)**

National Institute of Parasitic Diseases

Chinese Center for Disease Control and Prevention

207 Rui Jin Er Road

Shanghai 200025, People’s Republic of China

Tel.: +86 136 7161-6056

and

Department of Public Health and Epidemiology

Swiss Tropical Institute

Socinstrasse 57

CH-4051 Basel, Switzerland

Tel.: +41 61 284-8226

Fax: +41 61 284-8105

E-mail: peter.steinmann@unibas.ch

**Prof. Xiao-Nong Zhou (Epidemiologist and Deputy Director; Co-investigator)**

National Institute of Parasitic Diseases

Chinese Center for Disease Control and Prevention

207 Rui Jin Er Road

Shanghai 200025, People’s Republic of China

Tel.: +86 21 6473-8058

Fax: +86 21 6433-2670

E-mail: ipdzhouxn@sh163.net

**Prof. Jürg Utzinger (Epidemiologist; Co-investigator)**

Department of Public Health and Epidemiology

Swiss Tropical Institute

Socinstrasse 57

CH- 4002 Basel, Switzerland

Tel.: +41 61 284-8129

Fax: +41 61 284-8105

E-mail: juerg.utzinger@unibas.ch

**Introduction**

The Memorandum of Understanding (MoU) formalizing the research collaboration between the Swiss Tropical Institute (STI) in Basel and the National Institute of Parasitic Diseases (IPD) in Shanghai has been renewed in early 2008. Within this frame, a close research partnership between the research groups of Prof. Dr. Zhou XN at IPD and Prof. Dr. J. Utzinger at STI has been established. Supervised by Prof. Dr. J. Utzinger, P. Steinmann completed a Ph.D. in Epidemiology in November 2007. On 1 September 2008, Dr. P. Steinmann started his 2-year post-doctoral studies at IPD, jointly financed by the Swiss National Science Foundation (SNSF) and IPD.

The proposed randomized-controlled single-blind trial will establish the safety and efficacy of single- and triple doses of the standard anthelminthic drugs albendazole and mebendazole for the treatment of common soil-transmitted helminth (STH) infections in humans. Globally, STHs infect more than one billion people and combined, the burden due to these worms might be approaching that due to malaria. In China, these parasites are still common, especially in southern and western areas of the country where the socio-economic development and available sanitary infrastructure lack behind the standards achieved in the eastern parts of the country.

The proposed study will be implemented in southwest China in Nongyang village, Menghai county, Xishuangbanna prefecture, Yunnan province. The investigators have already worked in this village in 2006, and a randomized-controlled trial comparing single-dose albendazole with single-dose tribendimidine had been performed in a neighboring village in 2007 (EKBB No. 149/07) (Steinmann et al., 2008b). Thus, the team is well acquainted with the local conditions, and has established a close network of local contacts.

**Background**

Soil-transmitted helminths (STHs), namely *A. lumbricoides*, *T. trichiura* and the hookworms (*Ancylostoma duodenale* and *Necator americanus*), are pervasive in poor rural and deprived urban settings in tropical and subtropical environments (Bethony et al., 2006). Each of these worm species infects hundreds of millions of people, and many hosts harbor multiple species concurrently. STHs belong to the so-called neglected tropical diseases (NTDs). Whilst mortality attributable to STHs is low, these parasites are responsible for chronic and inconspicuous morbidity that has been largely underappreciated. Indeed, new research suggests that STH infections and, in an exacerbated way, multiparasitism result in significant morbidities and disabilities (Ezeamama et al., 2005b). Effects include iron-deficiency anemia, negative birth outcomes, malnutrition, and impaired physical and cognitive development (Ezeamama et al., 2005a). There is a paucity of high-quality data on the burden caused by STHs but the combined burden of disease (BoD) due to STH infections might be similar to that owing to malaria. Affected individuals cannot realize their full biological and intellectual potential. As a result, the economic perspectives of already disadvantaged societies are diminished, and the vicious cycle of poverty and disease sustained (Hotez et al., 2006).

Global STH control efforts currently rely on repeated mass drug administration (MDA) of single-dose anthelminthics targeting school-aged children or entire communities, depending on the estimated local prevalence (WHO, 2006). MDA is usually done without prior individual diagnosis. Only four drugs are included in the World Health Organization’s (WHO) Model List of Essential Medicines to treat human STHs, and global STH control currently relies on two of them, i.e the benzimidazoles albendazole and mebendazole (Keiser and Utzinger, 2008). Both drugs are marketed for decades already and have been extensively used worldwide. Yet, few systematic and standardized studies comparing their efficacy have been published, and reported values vary widely. However, evidence from a recent meta-analysis of published randomized, placebo-controlled drug efficacy studies indicates a marked superiority of albendazole, most notably with regard to curing hookworm infections. It was also found that previous studies are often hampered by the lack of a diagnostic ‘gold’ standard and poor study design issues (Keiser and Utzinger, 2008). Standard single-dose oral regimens usually result in high egg-count reduction rates for the common STHs, but cure rates can be low, particularly for hookworm and *T. trichiura*. Still, it is routinely assumed that albendazole and mebendazole can be used interchangeably, and currently more often than not the choice of the anthelminthic drug is based on availability, cost and policy rather than sound evidence.

China has fallen back behind other countries in the Western Pacific region regarding the control of common STHs. A national STH control programme has been established but regular MDA to vulnerable groups (pre-school and school-aged children, women of childbearing age) has not been achieved, not to mention a MDA programme covering the whole population at risk (Albonico et al., 2008).

The results of the proposed study will lead to a better understanding of the safety and efficacy of today’s standard anthelminthic treatment. The data will provide evidence to STH control programme managers regarding drug and dosage selection, and will inform the Chinese national helminth control programme regarding the drug-dosage combination of choice. Moreover, the data will directly benefit the local population since the results will be used in an upcoming evaluation of integrated intestinal helminth control tools investigating the additional benefit of health education and provision of improved sanitation over the effect of the most efficacious drug.

**Study aim and hypotheses**

*Aims:*

To assess the safety and efficacy of single-dose and triple-dose oral albendazole and mebendazole against common soil-transmitted helminth infections (i.e. *Ascaris lumbricoides*, hookworm and *Trichuris trichiura*) in a highly endemic setting in China, and to use this evidence-base to guide mass drug administration programs.

*Hypotheses:*

1. Both albendazole and mebendazole are safe for treating common soil-transmitted helminth infections in humans.
2. Single-dose oral albendazole and single-dose oral mebendazole are both highly active against *A. lumbricoides*, and result in high fecal egg count reduction rates for hookworm and *T. trichiura*.
3. Single-dose oral albendazole is more efficacious than single-dose oral mebendazole against hookworm infections.
4. Triple-dose oral albendazole and triple-dose oral mebendazole are significantly more efficacious against *T. trichiura* than single-dose oral regimens.

**Study design**

*Study type*

Open-label (single-blind) randomized-controlled trial with four arms:

1. Single-dose albendazole
2. Single-dose mebendazole
3. Triple-dose albendazole
4. Triple-dose mebendazole

*Primary outcome*

Cure rates of single-dose and triple-dose oral albendazole and mebendazole for treating common STH infections, especially hookworms.

*Secondary outcomes*

1. Safety of single-dose and triple-dose oral albendazole and mebendazole against common STH infections.
2. Egg-reduction rates of single-dose and triple-dose oral albendazole and mebendazole against common STH infections.

*Instruments*

Repeated quantitative fecal egg counts by the standard Kato-Katz thick smear method (Katz et al., 1972) and the recently developed, arguably more sensitive FLOTAC method (Cringoli, 2006; Utzinger et al., 2008).

Severity-graded assessment of treatment-related adverse effects using a questionnaire.

**Inclusion/Exclusion criteria**

*Inclusion criteria*

1. Age: ≥5 years old
2. Sex: males and females
3. Residency: Resident of Nongyang village in Menghai county, Yunnan province, People’s Republic of China
4. Registration, participation: Signing of written informed consent sheet (5-17 year olds: additional written approval by parents or legal guardians), willingness to comply with study procedures (stool submission, drug treatment, alcohol abstinence at day of treatment)
5. Absence of systemic or chronic diagnosed or perceivable illness as assessed by medical personnel upon enrolment and again at each day of treatment
6. Females: not pregnant (as verbally assessed by female medical personnel upon enrolment and again at each day of treatment)

*Exclusion criteria*

1. Age: <5 years
2. Residency: No permanent resident of Nongyang village in Menghai county, Yunnan province, People’s Republic of China
3. Registration, participation: No written informed consent sheet signed or parental approval not obtained, unwillingness to comply with all study procedures
4. Presence or suspicion of any abnormal medical condition which is known or suspected to interfere with anthelminthic treatment
5. Known hypersensitivity to any anthelminthic drugs
6. Recent history of anthelminthic treatment
7. Current or past (within 1 month) participation in any other medical trial
8. For females: Suspected or verified pregnancy

**Treatment and study procedures**

*Location*

Nongyang village in Menghai county, Xishuangbanna prefecture, Yunnan province, People’s Republic of China (Steinmann et al., 2008a; Steinmann et al., 2007).

*Time*

Preparation: August 2008-October 2008

Fieldwork, laboratory diagnosis: November 2008-February 2009

Data analysis and publication: March 2009-June 2009

*Information, enrolment*

The village authorities of Nongyang village will be informed about the study in detail by the study team. Subsequently, the resident population of Nongyang village will be briefed about the study by the village authorities. Upon initiation of the field work, teams of fieldworkers (composed of local health personnel and study team members) will visit all households in the village individually, provide detailed information about the planned study, answer arising questions exhaustively and obtain written informed consent to participate in the study from interested individuals and their legal guardians, if applicable (Appendix 1). Demographic data of participating individuals will also be collected (Appendix 2), and each participant will be assigned a unique identification number in ascending format (Format: XX-YY with X=family number and Y=individual number, starting with 01-01)

*Sample collection and diagnostic procedures for baseline parasitological survey*

Labeled stool sample collection containers will be handed out to interested participants upon signing the informed consent sheet. Participants will be invited to submit two large stool samples over consecutive days or within one week. Filled containers will be collected each morning between 6 a.m. and 9 a.m., and transferred to the laboratory at the Menghai county Center for Disease Control and Prevention.

All stool samples will processed within 6 h of collection. Two Kato-Katz thick smears will be prepared from each sample, and ~1 g of stool weighted and conserved in sodium acetate-acetic acid-formalin (SAF) solution for later analysis by the FLOTAC method. The Kato-Katz thick smear slides will be read within 45 min after preparation, and the SAF-conserved FLOTAC samples will be read within 12 weeks of collection. While the focus of the study is on common STHs (*A. lumbricoides*, hookworm, *T. trichiura*), additional helminths will be diagnosed whenever possible (*Taenia* spp. is known to be common in this community (Steinmann et al., 2008a)).

The total number of available diagnostic results will thus be: two Kato-Katz thick smears/sample, one FLOTAC apparatus with two different solutions/sample (total: three diagnostic test results/sample; six diagnostic test results at baseline). Kato-Katz thick smear slides and the FLOTAC are both read quantitatively.

*Randomization and drug dosage*

All participants with complete diagnostic results will be listed in ascending order according to their identification number, and the four different treatment options coded 1-4. A random sequence of 1s, 2s, 3s and 4s will then be generated, and aligned with the list of participants, thus assigning each individual a specific drug-dosage combination.

The drug dosage will be:

Daily albendazole dose: 400 mg (1 day or 3 days)

Daily mebendazole dose: 500 mg (1 day or 3 days)

For each participant, an identical envelope labeled with his/her name and containing the appropriate drugs according to the assigned treatment will be prepared.

*Treatment procedures, drug dosage and application*

Upon completion of the baseline parasitological survey, all participants with complete diagnostic results and ≥1 STH infection will be invited for treatment. They will be informed again about the treatment procedures, asked not to drink alcohol on treatment days, and to disclose their health and pregnancy status prior to treatment. On the days of treatment, teams of local health personnel and study team members will visit the participants individually in their houses after dinner time, enquire about their health and pregnancy status, and distribute the allocated drugs together with bottled water. Drug intake will be directly observed. This procedure will be repeated two times for those participants allocated to triple-dose treatment.

*Safety assessment:* All participants will be interviewed regarding adverse events of the treatment 24 h after drug application using a detailed semi-quantitative questionnaire providing a grading system for reported symptoms (Appendix 3). A medical doctor will be permanently available to the participants for 24 h after drug application for immediate attention to any adverse drug-related events which will be described in detail on an individual basis.

*Sample collection and diagnostic procedures for follow-up parasitological survey*

Two to three weeks post-treatment, labeled stool sample collection containers will be handed out to the treated study participants again. Participants will be invited to submit again two large stool samples over consecutive days or within one week. Filled containers will be collected each morning between 6 a.m. and 9 a.m., and transferred to the laboratory in the Menghai county Center for Disease Control and Prevention.

All stool samples will processed within 6 h of collection. Two Kato-Katz thick smears will be prepared from each sample, and ~1 g of stool weighted and conserved in SAF solution for later analysis by the FLOTAC method. The Kato-Katz thick smear slides will be read within 45 min after preparation, and the SAF-conserved FLOTAC samples will be read within 12 weeks of collection. While the focus of the study is on common STHs (*A. lumbricoides*, hookworm, *T. trichiura*), additional helminths will be diagnosed whenever possible (*Taenia* spp. is known to be common in this community (Steinmann et al., 2008a)).

The total number of available diagnostic results will thus be: Two Kato-Katz thick smears/sample, 1 FLOTAC apparatus with 2 different solutions/sample (total: 3 diagnostic test results/sample; 6 diagnostic test results at follow-up). Kato-Katz thick smear slides and the FLOTAC are both read quantitatively.

*End-of-study treatment*

At the completion of the follow-up parasitological survey, standard anthelminthic treatment (400 mg albendazole, single dose) will be offered to all participants still harboring STHs, to study participants not eligible for treatment within the study, and to all interested inhabitants of Nongyang village provided they are not pregnant, not suffering from systemic, chronic or specific illnesses preventing anthelminthic treatment, and do not take alcohol or other drugs known to interfere with benzimidazole drugs. Drugs for the treatment of additional helminth infections will be provided to individuals with a diagnosed infection.

**Risk/benefit assessment**

The study participants will benefit from free diagnosis and treatment of intestinal helminths. Adverse effects of anthelminthic treatment are uncommon. They include nausea, vomiting, and other transient gastro-intestinal symptoms. Severe adverse events are extremely rare.

**Data analysis**

The species-specific cure rates in individual study arms will be compared using the χ^2^ test. For each individual, the arithmetic mean number of eggs per gram stool (EPG) before and after treatment and for each helminth species will be calculated based on the egg counts resulting from the different diagnostic tests. Subsequently, the geometric mean EPG for the different study arms will be calculated, and the fecal egg count reduction test employed to compare the achieved specie-specific egg burden reductions.

**Sample size**

At least 370 individuals aged ≥5 years will be enrolled. Enrolment will be stopped once 400 individuals have signed up, even if additional volunteers are available. The calculated sample size is based on the following assumptions: A total of 176 individuals (44 in each of the four treatment arms) will be needed to demonstrate that single-dose albendazole is performing better than single-dose mebendazole for the cure of hookworm infections with 90% power using a 1-sided statistical test with an α-level of 0.05. The cure rates of albendazole and mebendazole are assumed to be 75% (76.4% in a recent meta-analysis of all published randomized placebo-controlled trials) and 45% (22.9%, multiplied by 2 to increase safety margin) (Keiser and Utzinger, 2008). To further increase the safety margin, the local prevalence of triple *A. lumbricoides*, *T. trichiura* and hookworm infections is assumed to be 60% (it was 76% during the study in 2006 (Steinmann et al., 2008a)), and the compliance is estimated to be 80%.

**Ethical considerations, data privacy, voluntariness, insurance**

Today, benzimidazole drugs are handed out to millions of people every year (Hotez et al., 2007), and medical ethics as well as the high burden resulting from STHs call for the consistent use of the most efficacious drug available. However, few rigorous studies assessing their efficacy have been published, and none are available which directly compare their safety and efficacy in a highly endemic setting and using a single-blind, randomized-controlled design (Keiser and Utzinger, 2008). In the frame of MDA campaigns, both drugs are administered to ≥2 year old individuals without previous diagnostic assessment or follow-up assessment of adverse events and treatment efficacy. The treatment of pregnant women is debated but pregnancy status is not commonly assessed before treatment (WHO, 2006).

All data collected within the frame of the study will be considered confidential and access restricted. During data analysis, individuals will only be identified using identification numbers, and neither names nor identification numbers will appear in any publications.

Participation in the study is strictly voluntary, and individuals not participating in the study will not experience any disadvantages. Full insurance coverage is provided by the institutional insurance covering the research activities of the STI.

**References**

Albonico, M., Allen, H., Chitsulo, L., Engels, D., Gabrielli, A.F., Savioli, L., 2008. Controlling soil-transmitted helminthiasis in pre-school-age children through preventive chemotherapy. PLoS Neglected Tropical Diseases 2, e126.

Bethony, J., Brooker, S., Albonico, M., Geiger, S.M., Loukas, A., Diemert, D., Hotez, P.J., 2006. Soil-transmitted helminth infections: ascariasis, trichuriasis, and hookworm. Lancet 367, 1521-1532.

Cringoli, G., 2006. FLOTAC, a novel apparatus for a multivalent faecal egg count technique. Parassitologia 48, 381-384.

Ezeamama, A.E., Friedman, J.F., Acosta, L.P., Bellinger, D.C., Langdon, G.C., Manalo, D.L., Olveda, R.M., Kurtis, J.D., McGarvey, S.T., 2005a. Helminth infection and cognitive impairment among Filipino children. Am J Trop Med Hyg 72, 540-548.

Ezeamama, A.E., Friedman, J.F., Olveda, R.M., Acosta, L.P., Kurtis, J.D., Mor, V., McGarvey, S.T., 2005b. Functional significance of low-intensity polyparasite helminth infections in anemia. J Infect Dis 192, 2160-2170.

Hotez, P., Raff, S., Fenwick, A., Richards, F., Jr., Molyneux, D.H., 2007. Recent progress in integrated neglected tropical disease control. Trends Parasitol 23, 511-514.

Hotez, P.J., Molyneux, D.H., Fenwick, A., Ottesen, E., Ehrlich Sachs, S., Sachs, J.D., 2006. Incorporating a rapid-impact package for neglected tropical diseases with programs for HIV/AIDS, tuberculosis, and malaria. PLoS Med 3, e102.

Katz, N., Chaves, A., Pellegrino, J., 1972. A simple device for quantitative stool thick-smear technique in schistosomiasis mansoni. Rev Inst Med Trop São Paulo 14, 397-400.

Keiser, J., Utzinger, J., 2008. Efficacy of current drugs against soil-transmitted helminth infections: systematic review and meta-analysis. J Am Med Assoc 299, 1937-1948.

Steinmann, P., Du, Z.W., Wang, L.B., Wang, X.Z., Jiang, J.Y., Li, L.H., Marti, H.P., Zhou, X.N., Utzinger, J., 2008a. Extensive multiparasitism in a village of Yunnan province, China, revealed by a suite of diagnostic methods. Am J Trop Med Hyg 78, 760-769.

Steinmann, P., Zhou, X.N., Du, Z.W., Jiang, J.Y., Wang, L.B., Wang, X.Z., Li, L.H., Marti, H., Utzinger, J., 2007. Occurrence of *Strongyloides stercoralis* in Yunnan province, China and comparison of diagnostic methods. PLoS Negl Trop Dis 1, e75.

Steinmann, P., Zhou, X.N., Du, Z.W., Jiang, J.Y., Xiao, S.H., Wu, Utzinger, J., 2008b. Tribendimidine for treatment of *Strongyloides stercoralis* and *Taenia* spp. infections: open label trial in China. PLoS Neglected Tropical Diseases, (under review).

Utzinger, J., Rinaldi, L., Lohourignon, L.K., Rohner, F., Zimmermann, M.B., Tschannen, A.B., N'Goran, E.K., Cringoli, G., 2008. FLOTAC: a new sensitive technique for the diagnosis of hookworm infections in humans. Trans R Soc Trop Med Hyg 102, 84-90.

WHO, 2006. Preventive chemotherapy in human helminthiasis: coordinated use of anthelminthic drugs in control interventions: a manual for health professionals and programme managers. World Health Organization, Geneva.

**Appendix 1: Study information and informed consent sheet** (will be translated into Chinese)

SAFETY AND EFFICACY OF SINGLE- AND TRIPLE-DOSE ALBENDAZOLE AND MEBENDAZOLE AGAINST

SOIL-TRANSMITTED HELMINTHS IN HUMANS:

OPEN-LABEL RANDOMIZED-CONTROLLED TRIAL IN CHINA

**This study is jointly undertaken by researchers from the following institutions:**

- Yunnan Institute for Parasitic Diseases (YIPD), China CDC, Simao, People’s Republic of China

- National Institute of Parasitic Diseases (IPD), China CDC, Shanghai, People’s Republic of China

- Swiss Tropical Institute (STI), Basel, Switzerland

**Principal investigators:** The following persons are directly responsible for the design and the implementation of the study: Dr. Peter Steinmann (IPD/STI), Dr. Du Zun-Wei (YIPD), Prof. Dr. Zhou Xiao-Nong (IPD), Prof. Dr. Jürg Utzinger (STI)

**Esteemed interested study participant!**

**Aim of the study**  Many people harbour worms in their intestine. These worms can cause a wide range of diseases and conditions ranging from mild and unnoticed to severe and debilitating. Among the main symptoms are intestinal problems, general weakness, anaemia and fatigue. Children and pregnant women suffer most from infections. Albendazole and mebendazole are two commonly used drugs to treat these infections. Both drugs are licensed for use in humans in China, and globally, millions of people are treated with these drugs every year. It is the aim of this study to compare the safety and efficacy of single- and triple doses of these two drugs.

**Study location, timeframe, recruitment and legal issues** The study will be performed in Nongyang village between November 2008 and February 2009. Interested inhabitants of Nongyang village aged ≥5 years who do not suffer from any other disease and are not pregnant are invited to participate in this study. A maximum of 400 participants will be recruited and all study procedures fully comply with the applicable Chinese laws and international guidelines.

**Procedures** Participants will be asked to provide two stool samples at the beginning of the study. These will then be checked for worm eggs. Infected individuals will then receive either a single or a triple dose of albendazole or mebendazole. The drug-dosage combination given to each participant will be assigned by chance and participants are not informed about the drug-dosage combination assigned to them (blinding). Participants will be asked about any adverse effects of the treatment after 1 day. Two-3 weeks after the treatment, another 2 stool samples will be collected and analyzed.

**Obligations** All study participants are obliged to fully comply with the study procedures, and follow the decisions of the study physician. The study physician must be informed about all health problems, pregnancy status, trial participation and drugs taken during or within 1 month prior to the study. The study team will inform you about relevant news regarding the study.

**Confidentiality:** All collected data are confidential. The results of the study may be published but no names or identifying pictures of individuals will appear in any publication without prior explicit consent. No personal data and diagnoses will be made available to other people, authorities or companies than those directly involved in the study and study supervision in a way that allows the identification of real persons.

**Benefits and risks:** Participants benefit from free diagnosis and treatment of intestinal helminth infections. The results of the study will further the planning and design of future treatment campaigns. Both drugs have an excellent safety record and adverse effects are rare. They include mostly mild gastro-intestinal and systemic symptoms. The treatment of pregnant women with both drugs is generally considered safe.

**Participation/withdrawal:** Participation in this study is entirely voluntary and you are free to withdraw from the study at any moment without further obligations.

**Costs/compensation:** Participants will not incur any costs by participating in this study. All damage incurred in the frame of this study is covered by a special insurance.

**Contact person for further questions and complaints:** Any questions or complaints can be directed at the fieldworkers who will try to answer them or transfer them to one of the principal investigators. You may also wish to contact directly Prof. Dr. Zhou Xiao-Nong, office: +86 21 64738058, mobile: 13916230620.

**I have read and understood this text and all my questions have been answered. I am aware of my rights and duties and I freely accept to participate in this study.**

**Date and signature of interested participant: Caretaker if <18 years old:**

**Date and signature fieldworker:**

**Appendix 2: Participation monitoring sheet**

| **Village: Nongyang** | | | |  |  | |  |  |  |  |  |  |
| --- | --- | --- | --- | --- | --- | --- | --- | --- | --- | --- | --- | --- |
| **Family name:__________________** | | | | | **Family ID:__________** | | |  |  |  |  |  |
|  | |  |  | |  | |  |  |  |  |  |  |
| **Family members** | | |  | |  | |  |  |  |  |  |  |
|  |  | |  | |  | |  |  |  |  |  |  |
|  | **Name, first name** | | **ID** | | **Year of birth** | **Sex** | **Participation** | **Sample A** | **Sample B** | **Treatment** | **Sample C** | **Sample D** |
|  |  |  |  |  |  | **M/F** | **N/Y (Signed)** |  |  |  |  |  |
| **1** |  | |  | |  |  |  |  |  |  |  |  |
| **2** |  | |  | |  |  |  |  |  |  |  |  |
| **3** |  | |  | |  |  |  |  |  |  |  |  |
| **4** |  | |  | |  |  |  |  |  |  |  |  |
| **5** |  | |  | |  |  |  |  |  |  |  |  |
| **6** |  | |  | |  |  |  |  |  |  |  |  |
| **7** |  | |  | |  |  |  |  |  |  |  |  |
| **8** |  | |  | |  |  |  |  |  |  |  |  |
| **9** |  | |  | |  |  |  |  |  |  |  |  |
| **10** |  | |  | |  |  |  |  |  |  |  |  |

**Appendix 3: Adverse event registration and grading sheet**
