# Supplementary figures and images for: Efficacy of Single-Dose and Triple-Dose Albendazole and Mebendazole against Soil-Transmitted Helminths and Taenia spp.: A Randomized Controlled Trial
Source: PLoS One. 2011 Sep 27;6(9):e25003. doi: 10.1371/journal.pone.0025003 (PMC3181256; doi:10.1371/journal.pone.0025003)

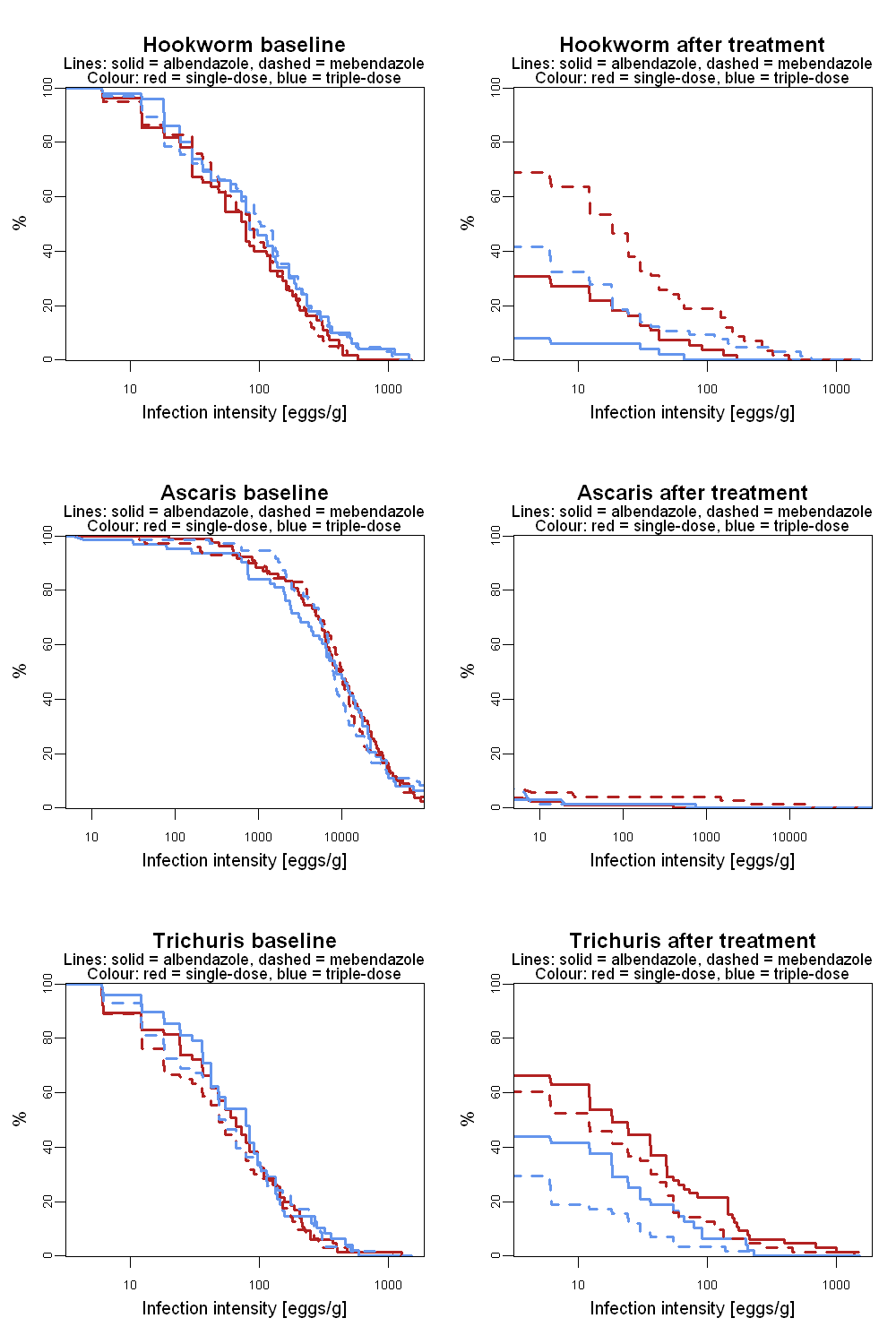

Supplement: Figure S1 — Frequency distribution of baseline EPGs and changes following treatment. (PNG) [file pone.0025003.s003.png]
