# Supplementary material for: Efficacy of Single-Dose and Triple-Dose Albendazole and Mebendazole against Soil-Transmitted Helminths and Taenia spp.: A Randomized Controlled Trial
Source: PLoS One. 2011 Sep 27;6(9):e25003. doi: 10.1371/journal.pone.0025003 (PMC3181256; doi:10.1371/journal.pone.0025003)
Supplement: Codes S1 — Codes to raw data of the trial. (DOC) [file pone.0025003.s005.doc]

Id: ID number (attention: there are data for all participants – limitation see “complete”)

sex: 0=female, 1=male

age: age in years

drug: A=albendazole, M=mebendazole

applications: 1=1 dose, 3=3 doses

complete: all samples and full treatment (I always use the “if complete=1” modification)

sample_a etc.: a, b are the 2 samples before treatment, d, e are the samples after treatment

asc_kk_a1 etc. Ascaris egg count in the Kato-Katz slide 1 (there were 2 slides for each sample)

asc: Ascaris

hw: hookworm

tri: Trichuris

tae: Taenia

asc_bl etc.: at least 1 Ascaris positive result at baseline

asc_eval etc.: at least 1 Ascaris positive result at evaluation

bl: baseline (before treatment)

eval: evaluation (after treatment)

asc_kk_int_bl_mean etc.: arithmetic mean Ascaris egg count at baseline (4 Kato-Katz slides from 2 samles)

asc_kk_int_eval_mean etc.: arithmetic mean Ascaris egg count at evaluation (4 Kato-Katz slides from 2 samples)

asc_kk_int_bl etc.: Ascaris baseline infection intensity classification according to WHO

asc_kk_int_eval etc.: Ascaris evaluation infection intensity classification according to WHO
